# Profile of physiotherapists working with soccer teams in South Africa

**DOI:** 10.4102/sajp.v79i1.1920

**Published:** 2023-10-19

**Authors:** Matthews Selomo, Maria E. Cochrane, Muhammad A. Dawood

**Affiliations:** 1 Department of Physiotherapy, Faculty of Health Care Sciences, Sefako Makgatho Health Sciences University, Pretoria, South Africa

**Keywords:** physiotherapy, profile, soccer, football, teams, education, South Africa

## Abstract

**Background:**

Soccer is one of the fastest growing sports in South Africa and the number of physiotherapists working with soccer teams has increased significantly. Despite increased appointments, very little is known regarding the demographic, education and work profiles of these physiotherapists.

**Objective:**

To determine the profiles of physiotherapists working with soccer teams in South Africa.

**Methods:**

A descriptive, cross-sectional study was used to collect data from physiotherapists employed with soccer teams. Physiotherapists who were employed on a part-time basis and not registered with the Health Professions Council of South Africa and who did not give consent were excluded. A total of 38 physiotherapists working with soccer teams participated in our study. A questionnaire was circulated, and participants were given 4 months to complete and submit it.

**Results:**

Results showed that participants had a mean age of 31.35 years and were employed for a mean time of 3.41 years. Most participants were African (89.48%) and worked with amateur soccer teams (52.63%). The education results indicated that 66.67% of participants held bachelor’s degrees. Postgraduate- and undergraduate education were used most frequently by participants to guide clinical decision-making. Job satisfaction was satisfactory, but they were not satisfied with their salaries.

**Conclusion:**

Our study is the first to investigate the profiles of physiotherapists working with soccer teams in South Africa. Demographic, education and work profiles for physiotherapists working with soccer teams were compiled, and the lack of information regarding the profiles of these physiotherapists was identified.

**Clinical implications:**

Extensive future research is needed to inform and train physiotherapists regarding the management of soccer teams.

## Introduction

Soccer is one of the fastest growing sports in South Africa (Mogajane, Du Plessis & Slabbert 2019). To ensure the successful growth of the sport, various sporting agencies, private companies and the government have invested funds in soccer teams – from grassroots levels to professional clubs (Draper et al. 2012). With the increase in funding, soccer teams have been able to appoint professional medical support, such as doctors, physiotherapists, fitness coaches and biokineticists in South Africa (Schokkaert 2014).

The dynamics of working with a sporting team differ from working with individual athletes (Duarte et al. 2012). Sporting teams comprise of individuals who need to cooperate collectively to achieve successful outcomes in a sport (Duarte et al. 2012). Within the team, everyone’s strengths must be harnessed to ensure optimal individual performance, without compromising the performance of the team (Duarte et al. 2012). There are many advantages for sportsmen and women who are participating as part of a team, such as improved social and psychological health (Anderson, Ottesen & Thing 2018), maintained fitness (Herzog 2018) and improved confidence/self-esteem (Allender, Cowburn & Foster 2006). Despite the benefits, participating in sports as part of a team can also have disadvantages. Theisen et al. (2013) found that an athlete in a sports team is significantly more likely to sustain overuse and traumatic injuries than individual athletes. The increased risk of injury is associated with the training and competition volume, which is generally much higher for athletes in team sports (Theisen et al. 2013). To ensure optimal health and fitness of a sports team and to minimise the risk of injury, the medical staff and team coach must be sufficiently trained and experienced (Dijkstra et al. 2014). It is therefore recommended that each sporting team consists of a head coach, performance director, sports physician, physiotherapist, nutritionist or dietician and psychologist (Dijkstra et al. 2014).

Physiotherapists working with sports teams, especially soccer, are responsible for injury prevention, fitness training, management of injuries once they occur, and determining whether players are ready for competitive play (Lifshitz 2012; Olsen et al. 2004; Oyeyemi et al. 2017; Reinking, Austin & Hayes 2007). Athletes participating in sports, specifically at highly competitive levels, have a very high expectation of physiotherapists when it comes to injury management and rehabilitation (Quartey, Afidemenyo & Kwakye 2019). Although the roles of physiotherapists in soccer teams have been established, very little is known about the profiles of these physiotherapists in South Africa.

All physiotherapy graduates have a basic understanding of sports rehabilitation, as it is part of undergraduate training (Health Professions Council of South Africa [HPCSA]). The basic undergraduate training includes training of students to fulfil the various roles described earlier and to work collaboratively, but none of the universities in South Africa currently prepares students specifically for working with sports teams. A recent study in Spain indicated that approximately 79% of students enter the physiotherapy profession because of its close relationship with sports (Fuente-Vidal et al. 2021). However, despite the interest in sport, physiotherapy students are not required to work with sports teams at an undergraduate level (nationally or globally) (World Physiotherapy Education Framework). It is therefore postulated that lack of training of students to work with sports teams is not due to the inabilities of the training institutions, but rather due to the dearth of information regarding the profiles of these physiotherapists. Our study, therefore, aimed to determine the demographic, education and work profiles of physiotherapists working with soccer teams. The information presented in our study could be used by training institutions to ensure that students are prepared and equipped to work in a soccer team environment. The results can also be used by physiotherapy students and physiotherapists to make informed decisions prior to joining soccer teams as health service providers.

## Methods

A descriptive, cross-sectional questionnaire-based study was used. Questionnaires were distributed to participants both electronically and in paper-based form. Online surveys are effective for collecting data from a large geographic sample and it minimises the risk of information getting lost in the postal system (Lefever, Dal & Matthiasdottir 2006). Participants in online surveys have also been observed to complete and return the questionnaires in a shorter timeframe than participants who complete hard-copy (paper-based) questionnaires (Lefever et al. 2006). However, in South Africa, it is estimated that only 54% of the population has access to a stable Internet connection (The World Factbook). Therefore, hard-copy (paper-based) questionnaires were also prepared and distributed to participants who did not have access to the Internet.

For the distribution of online questionnaires, SurveyMonkey cloud-based software was used. All the information gathered from SurveyMonkey was confidential, as participants in research projects’ names are never revealed to the survey developer. Any identifying information that was provided by participants was coded, to ensure anonymity of the participants. To ensure validity and reliability of the different methods of questionnaire distribution, a pilot study was conducted with five physiotherapists who were not affiliated with soccer teams. All five physiotherapists agreed that the questionnaire was uploaded in its entirety on SurveyMonkey, without errors; that the SurveyMonkey web link worked; that multiple attempts to complete the same questionnaire after submission was blocked; and that the online questionnaire was easy to complete. The data obtained from the pilot study were not used in the final data analysis of our study.

All physiotherapists working with soccer teams in South Africa were targeted for inclusion, regardless of the level of teams’ participation. There are approximately 45 structured soccer teams with dedicated physiotherapists in the country (i.e., the physiotherapists had signed contracts with the soccer teams). The physiotherapists were identified through the South African Football Association and the South African Society of Physiotherapists.

Using the Raosoft^®^ sample size calculator, a total of 41 physiotherapists had to be recruited to ensure a confidence level of 95%. A response rate of 60% was acceptable to the authors (Fincham 2008). After the participant recruitment and data collection procedures were executed, 38 physiotherapists returned questionnaires. The physiotherapists who did not complete the questionnaires were contacted but did not provide reasons for non-participation.

The physiotherapists recruited for participation were tested against the following eligibility criteria.

Inclusion:

- All physiotherapists (male and female of all ages) had to be registered with the HPCSA and had to be working with soccer teams in South Africa.
- The physiotherapists had to be dedicated team physiotherapists employed by a soccer team. The physiotherapists had to have a signed contract with a soccer team but may have been employed elsewhere as well.
- Physiotherapists recruited for participation were requested to provide voluntary written informed consent.

Physiotherapists were excluded from participation if they were not registered with the HPCSA, if they were working with soccer teams on a voluntary basis, and if they did not provide consent.

The participatory level of the soccer teams (i.e., amateur, professional, etc.) was not considered.

### Data collection

Prior to the onset of our study, ethical clearance was obtained. Participants were given 4 months to complete and return the questionnaires via the SurveyMonkey link, after which access to the website was terminated. Once participants had completed the questionnaire and submitted their answers, access to the questionnaire was terminated as well, to avoid duplication of information. An automated electronic reminder to complete the questionnaire was circulated to participants via the SurveyMonkey website on a weekly basis. Once the participants completed the questionnaire, the reminders were automatically ceased by the website.

Participants who did not have access to the Internet received the information brochure, informed consent and questionnaire via postal arrangements made with each potential participant on an individual basis. Only 2 of the 38 participants who participated in the study requested hard-copy (paper-based) questionnaires. A short message service (SMS) or a WhatsApp message was sent on a weekly basis to remind the participants to complete the questionnaire.

**FIGURE 1:**
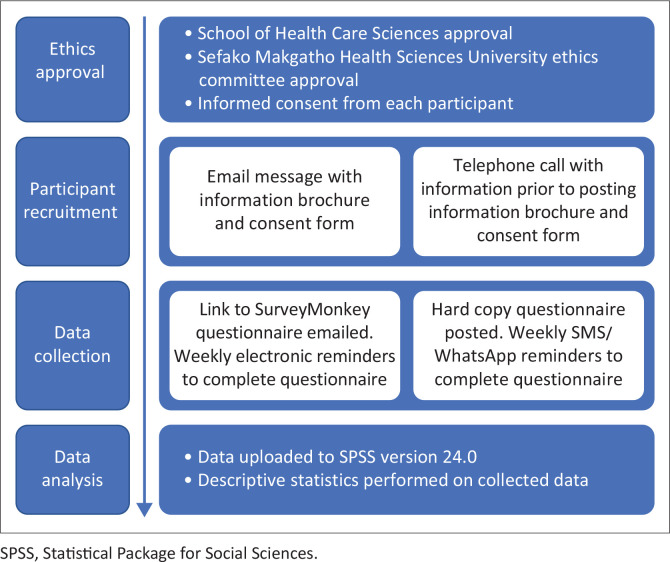
Data collection procedure.

A summary of the data collection procedure can be found in Figure 1.

### Data collection tool

The questionnaire that was developed for data collection consisted of two previously validated questionnaires, namely, the Physical Therapy Profile Questionnaire (PTPQ) and the Job Satisfaction Survey. The PTPQ was designed to establish a profile of physiotherapists who are employed in clinical work areas (public and private institutions), university settings and research settings. It is the only validated questionnaire available that specifically relates to physiotherapy, which is why it was selected for use. The face and content validity of the questionnaire have been established on a 5-point rating scale (1: lowest and 5: highest). The PTPQ has good face and content validity for usability (4.43), ease of administration (4.86), comprehensiveness (4.71) and format (4.43) (Dizon, Grimmer-Somers & Kumar 2011).

To determine the job satisfaction of physiotherapists working with soccer teams, the Job Satisfaction Survey was used. The survey was originally developed for use in the social sector but has since been used in all sectors (including healthcare) (Van Saane et al. 2003). The content validity of the survey was found to be high, but the discriminant validity was shown to be moderate (this is because the tool is not very responsive to change) (Van Saane et al. 2003). We therefore recommended that this tool is more suitable for once off use, than for testing over a period (which is how it was administered in our study).

### Data analysis

The collected data were analysed using the Statistical Package for Social Sciences (SPSS) version 24.0. Descriptive statistics, namely, the means, medians, standard deviations, frequencies and ranges, were used to determine the profiles of physiotherapists working with soccer teams in South Africa.

### Ethical considerations

Ethical clearance to conduct our study was obtained from the Sefako Makgatho Health Sciences Research and Ethics Committee (No. SMUREC/H/303/2017:PG). Eligible participants were contacted and sent an information brochure and consent forms. Once the informed consent documents were received from the participants, a link to the SurveyMonkey questionnaire was circulated to them.

## Results

The results are categorised into three main components, namely, demographic results, education or qualification results and work or education-related results. The results for each category are displayed as infographics.

### Demographic profiles

**FIGURE 2:**
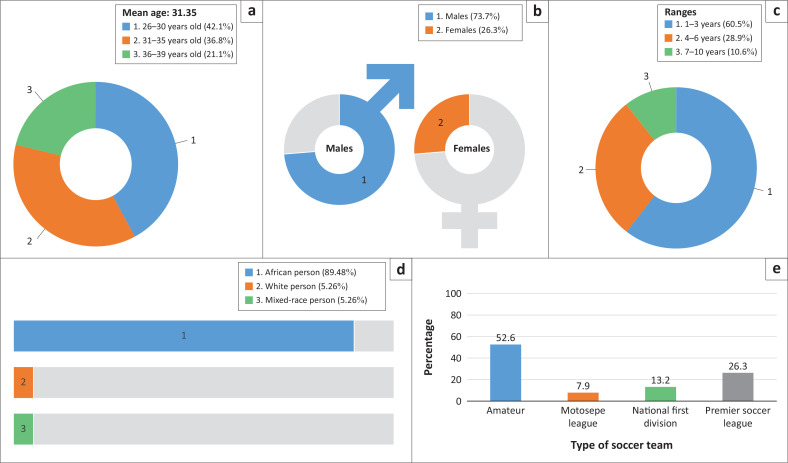
Demographic profile of physiotherapists working with soccer teams. (a) Age, (b) gender, (c) years of employment (years of employment with the soccer team), (d) race, and (e) place of employment (type of soccer team physiotherapists is employed at).

The mean age of participants was 31.35 years (standard deviation [s.d.] = 4.138). Most participants were male (*n* = 28) and African (*n* = 34). They were employed by their soccer teams for a period between 1 and 10 years. Twenty participants worked with amateur soccer teams, 3 worked with Motsepe League (ML) teams; 5 worked with National First Division (NFD) soccer teams and 10 worked with Premier Soccer League (PSL) teams (Figure 2).

### Education or qualification profiles

**FIGURE 3:**
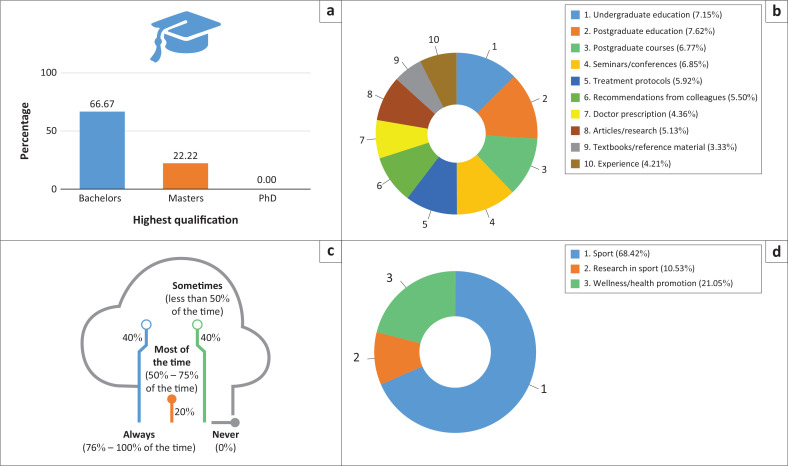
Education or qualification profile of physiotherapists working with soccer teams. (a) Highest education, (b) factors influencing clinical decision making, (c) Internet usage in clinical decision making, and (d) special interests of physiotherapists.

The majority of participants (*n* = 35) had a bachelor’s degree, and three participants were studying towards a master’s degree. During daily decision-making, most participants relied on their postgraduate and undergraduate training, with some using information from seminars and conferences and (non-degree) postgraduate courses. When using the Internet to assist with decision-making, 40% indicated that they always consult the Internet, while 40% indicated that they only sometimes (less than 50% of the time) use the Internet. It was interesting to note that very few participants (*n* = 4) showed an interest in conducting research in sport (Figure 3).

### Work or employment profiles

**FIGURE 4:**
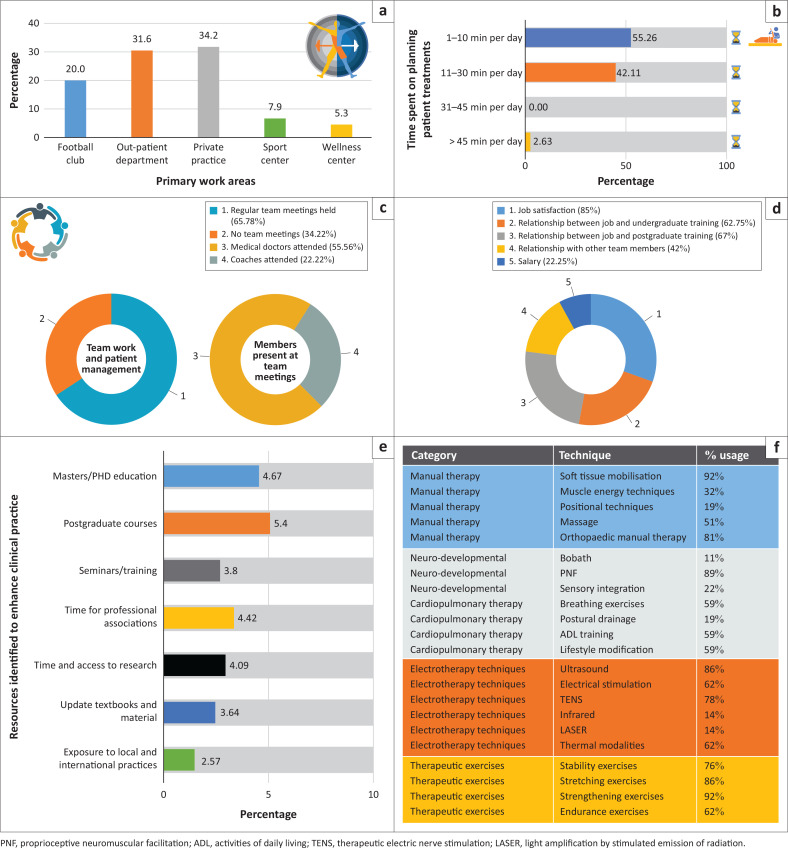
Work or employment profiles of physiotherapists working with soccer teams. (a) Primary (physical) work areas, (b) Time spend on planning patient treatment, (c) team work and patient management and members present at team meetings, (d) job satisfaction, (e) resources identified to enhance clinical practice, and (f) frequently used clinical practices.

Despite working with soccer teams, most participants indicated that they treat the soccer players at private practices or in outpatient departments. When asked regarding planning of patient treatments, 75% of participants indicated that they spend time planning treatments, and the majority (55.26%) indicated that they spend between 1 and 10 min per day on planning. To determine the teamwork that underlies patient management in soccer teams, participants were asked how frequently team meetings were held to discuss patients’ health management. Twenty-five participants (65.78%) indicated that they held team meetings to discuss patients’ health management, but the other 34.22% of participants did not. The participants who indicated that they had team meetings were asked to indicate the team members present at these meetings. Medical doctors (team physicians) were present at 55.56% of the meetings and coaches were present at 22.22% of the meetings. No other health professionals or soccer team stakeholders were present at health management meetings. Participants were also asked to indicate their satisfaction with their current employment. Most participants were satisfied with their work, the relationship between their work and studies, and their relationships with colleagues at work. The results also indicated that most participants were not satisfied with the salaries they earned (Figure 4).

Lastly, to get a comprehensive overview of the work-related aspects of the profiles, participants were asked to indicate the clinical practices that they use most frequently when managing soccer players. The participants indicated that they use strengthening exercises, proprioceptive neuromusculoskeletal facilitation, stretching exercises, soft tissue mobilisation and ultrasound most frequently (Figure 4).

## Discussion

### Demographic profiles

To determine the demographic profile of physiotherapists working with soccer teams in South Africa, the following variables were examined: age, gender, race, number of years working with the soccer team and the type of team that the physiotherapists were working for. The participants ranged between the ages of 26 and 39 years (mean = 31.35), which is relatively young as the documented working ages in South Africa range between 15 and 65 years (mean = 40) (Work and Labour Force, South Africa 2019). The average working age of physiotherapists in South Africa has not been established. Demographic information that was obtained through extensive literature searches indicated team physiotherapists’ ages to range between 28.5 and 33.5 years, which is similar to our results (Heaney 2006; McKenna, Delaney & Phillips 2002; Scott & Malcolm 2015; Silva et al. 2011). A possible reason for the relatively young ages of physiotherapists working with soccer teams could be the frequent travel demands imposed on them (Samuels 2012). Samuels (2012) found that sports teams and their supporting medical staff who travel frequently do not only suffer from jet lag (when travelling between time zones) but also suffer from travel fatigue. We further found that younger medical staff and sports teams recover quicker from the effects of travel fatigue than older people (Samuels 2012).

About 89.4% of participants were African. Our results are similar to the findings from the Transformation Committee Eminent Persons Group, which indicated that soccer is the only sport that met the transformation goals across the different domains (such as racial representation in the sport, employment equity, senior team management transformation, women on boards, etc.) (Transformation Status Overview). The results also indicate that most participants were employed with the team for an average time of 3.41 years (range 1–10 years). From the available studies, the time of employment of South African physiotherapists with a team is relatively short. McKenna et al. (2002) and Scott and Malcolm (2015) indicate an average time of physiotherapists’ employment as 8.5 and 11.2 years, respectively. However, Silva et al. (2011) found that the average time of employment of physiotherapists with a soccer team was only 2.5 years. There is no indication of possible reasons why physiotherapists’ employment with sports teams (including soccer teams) is relatively short. In addition, no information is available regarding the frequency with which teams change healthcare practitioners or physiotherapists. In an interview, a sevens rugby team sports physiotherapist speculated that physiotherapists working with elite sporting teams may change frequently because they move between teams (Chan 2016). Further research to investigate the frequency with which sporting teams change healthcare practitioners and reasons why physiotherapists are generally employed with teams for a short period of time is recommended to provide clarity on the subject.

### Education or qualification profiles

Most participants (66.67%) had obtained a bachelor’s degree in physiotherapy as their highest qualification. Approximately 22% of the participants held a master’s degree or were studying towards obtaining one, and none of the participants had PhD qualifications. Our findings differ from other studies where the majority of physiotherapists working with soccer teams have a master’s degree (McKenna et al. 2002; Meurer, Silva & Baroni 2017; Scott & Malcolm 2015). The low number of master’s degree holders in South Africa might be a result of the limited financial benefits associated with the degree (Cobbing et al. 2017). Additional barriers to obtaining formal postgraduate qualifications (such as a master’s degree and PhD) are found to include concerns regarding the ability to complete the degree, family commitments and lack of motivation, time and employer support (Cobbing et al. 2017). Cobbing et al. (2017) identified that most postgraduate students only pursue a postgraduate degree if it is a compulsory requirement at the place of employment or if a person wants to move to a different field (within the physiotherapy profession). Currently, it is not a requirement for sports physiotherapists working with soccer teams to be in possession of a postgraduate degree. In addition, sports physiotherapists travel frequently, thereby increasing the chances that time limitations will influence their decision to pursue a postgraduate qualification (Samuels 2012).

Thirty-eight point eighty nine percent of participants attended postgraduate courses or obtained postgraduate certificates. This finding is similar to other studies (McKenna et al. 2002; Meurer et al. 2017; Scott & Malcolm 2015). Although our results are encouraging (in that there is an interest in attending postgraduate courses), it is not clear whether the attendance of postgraduate courses is a result of interest in life-long learning or because it is mandated.

A part of the daily requirements of a physiotherapist’s role is that they are required to make decisions. The participants were asked to indicate what aspects of education, or their qualifications, informed their daily decision-making. Postgraduate education was indicated as the most frequent source on which decision-making is based. This result was unexpected, as most of our study population did not have a postgraduate qualification. Although reasons for giving the answer can only be speculated about, it might be that the participants misunderstood the term ‘postgraduate education’ and selected this option if they used postgraduate education and postgraduate courses as the main source on which they base their daily decision-making. This possible misinterpretation of the question indicates the need for establishing questionnaires that are developed specifically within a South African context.

Undergraduate education was indicated as the second most used source on which decision-making is based. Although there is no evidence specifically pertaining to decision-making in sports team physiotherapists, this result is similar to other studies. Holder, Wallin and Heiwe (2013) report that physiotherapists use their basic education as the main factor in clinical decision-making. Using formal education as a basis for decision-making assists with the implementation of evidence-based practice (Scurlock-Evans, Upton & Upton 2014). Scurlock-Evans et al. (2014) in their systematic review to investigate the implementation of evidence-based practice in physiotherapy found that the implementation of evidence-based practice is sporadic and inconsistent (Scurlock-Evans et al. 2014). The results obtained in the systematic review by Scurlock-Evans et al. (2014) are very similar to our results. We also found that physiotherapists prefer to fall back on basic education during clinical decision-making; that physiotherapists will use treatment and rehabilitation protocols for decision-making when they are available (protocols are indicated as the fifth most used source of decision-making); and that very few physiotherapists use textbooks or reference material for decision-making (textbooks and reference material were the least likely sources of decision-making). Personal experience was indicated as the second least likely source to use for decision-making. This result is encouraging, as physiotherapists who use experience as a main source for decision-making are not likely to participate in evidence-based treatment (Condon et al. 2016; Scurlock-Evans et al. 2014).

Using the Internet as a source for decision-making was asked in a separate question, as various factors (such as access to the Internet) affected the outcome of the question. The participants indicated that they had frequent access to the Internet, apart from two participants (5.26%) who indicated that they did not have frequent Internet access. About 40% of the participants indicated that they consult Internet resources 76% – 100% of the time for clinical decision-making, and 40% reported that they use the Internet less than 50% of the time for decision-making. Fell, Burnham and Dockery (2013) found that physiotherapists who used the Internet as a source for decision-making preferred journal articles to general Internet resources. The study by Fell et al. (2013) was conducted in the United States, where access to the Internet and journal articles is higher than in South Africa. In South Africa, access to journal articles is generally limited to freely available content or open access articles (Czerniewicz & Goodier 2014). Only registered students at universities have access to paid articles through the institutions’ libraries. This could be a reason why some of the participants used the Internet frequently for clinical decision-making, and some participants did not use the Internet frequently at all.

The last component that was investigated to determine the education or qualification profiles of sports physiotherapists working with soccer teams in South Africa was their areas of special interest. Not surprisingly, most participants (68.42%) indicated that their special interests were in sports. Eight participants (21.05%) indicated that their special interest was in wellness and health promotion. As one of the core competencies expected of sports physiotherapists, health promotion is often overlooked, as it does not fall within the population’s expectations of physiotherapy services (Taukobong et al. 2013). It is encouraging that there is an interest among sports physiotherapists working with soccer teams to promote health and well-being in this population. Very few (10.53%) of the participants indicated that they are interested in research in sports. This may again be attributed to the lack of incentive to participate in postgraduate education (and by extension, in research activities) (Cobbing et al. 2017).

### Work or employment profiles

To our knowledge (and after extensive literature searches), no information could be found to indicate the physical areas in which sports physiotherapists work with players in soccer teams (or any other team). Our results indicate that most physiotherapists (34.2%) who work with soccer teams in South Africa conduct the patients’ assessments and treatments (and rehabilitation) in a private practice setting. Other settings indicated for the conduction of patient assessment and treatment were outpatient departments (31.6%), at the soccer clubs (20%), in a sports centre (7.9%) and in wellness centres (5.3%). Interestingly, no patient assessments and treatments are conducted on the field side. This could be because field side management is usually brief in nature, and only non-extensive assessments and fast decision-making are used to determine whether a soccer player can continue to play or if further (more extensive) management is required (Volpi et al. 2011).

In addition to where the physical work was performed, participants were asked to indicate how much time they spend on planning patient treatments. Most participants (55.26%) indicated that they spend between 1 min and 10 min per day planning treatments. Twenty-five percent of the participants indicated that they do not spend any time on planning patient treatments. Very little published information is available to indicate the ideal amount of time a physiotherapist should spend on planning patient treatments. Stevens et al. (2017) found that patients’ goals (for the outcome of the physiotherapy intervention) should determine the amount of time spent on planning patient treatments. It could be argued that the goal of the sports physiotherapist is to return a player to participation in the sport, irrespective of the injury, which might be a reason for the lack of treatment planning reported. Additional research to investigate the ideal amount of time that should be spent on planning patient treatments is needed to conclude our findings.

Physiotherapists are not the only role-players in the management of soccer players in teams. Teamwork is important to ensure the optimal performance of the team and to minimise the risk of injury (Dijkstra et al. 2014). Dijkstra et al. (2014) recommended that each sporting team should consist of a head coach, performance director, sports physician, physiotherapist, nutritionist or dietician and a psychologist; 65.78% of the participants indicated that they were involved in regular team meetings to discuss the health and well-being of the team. In these meetings, medial officers (usually the team physician), coaches and the physiotherapists were involved, but there were no other members of the multidisciplinary team in team meetings. The other participants (34.22%) indicated that they do not participate in frequent team meetings. The lack of team meetings will not only negatively affect the soccer teams’ health but will also undermine the competencies expected of sports physiotherapists. Soccer teams will lose out on the benefits of a full socioecological approach to injury prevention if frequent team meetings are not held (Bolling et al. 2018). The physiotherapists will also not have the opportunity to participate in team management to the full extent that will benefit the team (Malcolm 2006; McEwan & Taylor 2010). Because of the positive influence that a multidisciplinary team approach to soccer team management can have, it is recommended that all the healthcare providers and the coaches of soccer teams meet frequently to ensure optimal performance of the team (Longo et al. 2019).

An integral part of any employment is satisfaction with the job. Participants indicated that they are satisfied with their jobs, but most participants indicated that they are not satisfied with the salaries they earn. Numerous studies into job satisfaction in physiotherapy have been conducted, but none of the published research specifically investigated the satisfaction of physiotherapists working with soccer teams. Studying job satisfaction is important as it is directly linked to productivity as well as to personal well-being (Aziri 2011). Job dissatisfaction, on the other hand, may bring about a lack of productivity in a workplace and may result in poor work ethics, lateness and absenteeism (Markovits, Van Dick & Davis 2007). Bayzid et al. (2019) conducted a job satisfaction study wherein two-thirds of the physiotherapists (who were based in private practices) reported satisfaction with their working environments, leave policies and job security. However, more than half of the physiotherapists reported dissatisfaction with their salary structures, the promotion system at the place of employment and work-related stress (Bayzid et al. 2019). Additionally, Lu, While and Barriball (2005) and Singh and Jain (2013) indicate that individuals’ expectations from the job and the degree of motivation an employee also contributes to job satisfaction, or dissatisfaction. Bayzid et al. (2019) also showed that there was no significant relationship between the sexes, age and job satisfaction but found that there is a significant relationship between job satisfaction and salary. All our participants indicated some degree of dissatisfaction with their salaries.

The final aspect of work or employment that was studied was the frequently used clinical practices of the physiotherapists working with soccer teams in South Africa. The most frequently used clinical practices were soft tissue mobilisation (92%), strengthening exercises (92%), proprioceptive neuromuscular facilitation (89%), stretching exercises (86%), ultrasound therapy (86%), orthopaedic manual therapy (81%) and stability exercises (76%).

Stretching, strengthening and neuromuscular warm-up have been shown to be successful in injury prevention in soccer players (Herman et al. 2012; Read et al. 2018). In the management of acute injuries, various physiotherapy modalities are used to ensure optimal recovery and speedy return to play. These modalities include strengthening (Charlton et al. 2017; Quinn 2010) and stability training (Michaelidis & Koumantakis 2014). Ultrasound therapy is used to increase healing in acute injuries and to decrease time off the field (Van Den Bekerom et al. 2013). During rehabilitation of chronic or overuse injuries, strengthening, stretching, proprioceptive neuromuscular facilitation and stability training are used to ensure the optimal outcome of the injury and to prevent recurrent injuries (Bleakly, Glasgow & MacAuley 2012; Dhillon, Dhillon & Dhillon 2017; Taylor et al. 2009). Lastly, the most frequently used clinical practices are also used to enhance performance in soccer players. Again, stretching, strengthening, stability training, soft tissue mobilisation and proprioceptive neuromuscular facilitation are frequently used to ensure that soccer players’ performance in the team is enhanced (Akbari et al. 2018; Kobal et al. 2017; Jordan et al. 2012; Jovanovic et al. 2011).

## Conclusion

Our study was the first to investigate the profiles of physiotherapists working with soccer teams in South Africa. Demographic, education/qualification and work or employment profiles for physiotherapists working with soccer teams were compiled, and the lack of information (locally and globally) regarding the profiles of physiotherapists working with soccer teams was identified. Although our results give an indication of the profiles of these physiotherapists, extensive future research into physiotherapists working with soccer teams (and teams from different sporting codes) will have to be performed, so that physiotherapists can be adequately informed and trained regarding the management of sports teams.
